# Vitamin D status in post-medieval Northern England: Insights from dental histology and enamel peptide analysis at Coach Lane, North Shields (AD 1711–1857)

**DOI:** 10.1371/journal.pone.0296203

**Published:** 2024-01-31

**Authors:** Anne Marie E. Snoddy, Heidi Shaw, Sophie Newman, Justyna J. Miszkiewicz, Nicolas A. Stewart, Tina Jakob, Hallie Buckley, Anwen Caffell, Rebecca Gowland

**Affiliations:** 1 Department of Anatomy, University of Otago, Dunedin, Otago, New Zealand; 2 Department of Archaeology, Durham University, Durham, United Kingdom; 3 School of History, Classics and Archaeology, University of Edinburgh, Edinburgh, United Kingdom; 4 School of Social Science, University of Queensland, Brisbane, Queensland, Australia; 5 School of Applied Sciences, University of Brighton, Brighton, United Kingdom; UNCG: University of North Carolina at Greensboro, UNITED STATES

## Abstract

**Objectives:**

The post-medieval period in Europe saw a dramatic increase in metabolic bone disease related to vitamin D deficiency (VDD). Recent paleopathological work has utilized interglobular dentin (IGD) as a proxy for poor vitamin D status during development, while enamel peptide analysis allows the identification of chromosomal sex in non-adult remains. Here we explore the relationship between sex, the presence of IGD, and macroscopic markers of VDD in an industrial era assemblage from Northeast England.

**Materials and methods:**

25 individuals (9 females, 9 males, 9 unknown sex) from the cemetery site at Coach Lane, North Shields (1711–1857) were selected for paleopathological analysis, histological assessment of IGD, and enamel peptide determination of chromosomal sex.

**Results:**

Ground tooth sections from 21 individuals were of suitable quality for detection of IGD, and enamel peptide analysis confirmed the chromosomal sex of ten individuals. Sixteen individuals (76.1%) exhibited ≥1 episode of IGD. Nine of these (42.8%) exhibited >1 episode and four (19%) exhibited ≥4 episodes in regular intervals. Male sex was significantly associated with the presence of IGD (*p* = 0.0351; 100% males vs. 54.5% females). Females were more likely to exhibit macroscopic evidence of VDD (45.5% females vs 30% males) but this was not statistically significant.

**Discussion and conclusions:**

Periods of poor mineral metabolism during childhood appear much more prevalent at Coach Lane than macroscopic evidence suggests. Evidence of seasonal IGD episodes indicates that northern latitude played a major role in poor VD status in the Northeast of England. The significant association of IGD with male sex may be due to sex-related differences in dentinal mineralization or a higher risk of poor VD status in males aged <5 years. More work is needed to establish an evidence-based threshold for pathological levels of IGD before the presence of this feature can confidently be used as a biomarker for poor VD status.

## Introduction

The post-medieval period (16^th^-19^th^ centuries AD) in England was characterized by increasing urbanization and a shift away from an agrarian economy towards manufacturing [1, 2]. While the British Empire grew in wealth and power, social inequality increased dramatically, observed in decreasing life expectancy of the laboring classes compared with the wealthy [3]. By the 19th century, large portions of the population, including young children, lived in overcrowded and unsanitary conditions, and worked long hours in unventilated spaces for very little remuneration [4]. Diseases associated with poor nutrition, poor sanitation, and crowded living conditions were rife in urban centers, particularly among the lower classes [5]. Recent advances in archaeological science provide the means to explore the physical embodiment of industrialization in detail, including the relationship between gendered cultural practices, biological sex, and disease during this period.

In the popular imagination, 19^th^ century England is associated with sickly, rachitic children, largely due to the works of Victorian novelists who brought enduring awareness to the physical consequences of poverty in Britain’s urban manufacturing centers (e.g. *Oliver Twist*, *Mary Barton*, *North and South*). Although much of the historical and bioarchaeological literature on rickets in Industrial England has focused on southern cities such as London [6–9], many of England’s major centers of industry were in the North [10, 11]. Coal mining, textile and ceramic production, and shipbuilding are just a few of the industries underpinning the Northern urban centers of Newcastle upon Tyne, Manchester, Leeds, and Sheffield, and each of these were associated with satellite manufacturing towns [11, 12]. In his 1855 medical survey, Hungarian physician Augustus Merei found rickets to be “common” among the laboring classes of Newcastle, and in Manchester and its satellite of Stockport. These findings were mirrored in a later 1889 British Medical Association study, which identified the Northeast as a focal zone for rickets [13–15]. Recent work [3, 16] has uncovered skeletal evidence for rickets in 19th century Northern English assemblages, with Newman and colleagues’ comparative study showing a higher prevalence of metabolic bone disease in cemetery assemblages from the Northeast of England than in those from London [16]. The prevalence of childhood rickets appears to be positively correlated with urbanization and lower socioeconomic status in the post-medieval period, suggesting that poor vitamin D status was a consequence of industrialization in the 18th and 19th centuries [17].

Understanding the epidemiological profile of vitamin D deficiency (VDD) in Northeast England during the 18^th^ and 19^th^ centuries has important implications beyond the identification of the metabolic bone disease it causes. Vitamin D status is closely related to the risk of developing other diseases or health conditions [18], which can be predicted based on an individual’s age and sex [19]. Vitamin D status is also tied to gendered work practices, which changed dramatically during the industrial period with the first large-scale employment of women in factories [20]. The age and sex distribution of VDD within a population, therefore, has important implications for understanding biological and cultural influences of VDD prevalence within a population.

Macroscopic skeletal lesions indicative of rickets have been previously reported for post-medieval industrial human remains recovered from Coach Lane, North Shields (AD 1711–1857) in Northeast England [3, 16, 21]. The true magnitude and distribution of poor vitamin D status in this assemblage cannot be known using macroscopic analysis alone, as this will only capture individuals of suitable preservation and with severe disease manifestations [22]. This study aims to build on previous analyses of rickets during this period through histological and new X- and Y-chromosome linked enamel peptide analysis of teeth from the Coach Lane assemblage, which have the power to provide higher resolution data on the distribution of disease. Previous bioarchaeological work on this site has been unable to explore the relationship between vitamin D deficiency during growth and sex due to the problems associated with sex estimation from non-adult remains.

### Vitamin D deficiency in archaeological human remains

The detection of VDD in archaeological human assemblages can provide a powerful window on health in the past [23, 24]. VDD has adverse effects on mineral metabolism [25, 26]. Physical manifestations of VDD and subclinical vitamin D insufficiency (VDI) span a spectrum in which diagnostic skeletal lesions are on the extreme end [22]. Furthermore, the active metabolite of vitamin D (1,25[OH]2D3) plays a key role in regulating several physiological processes outside of bone homeostasis and growth, including immune functions [27, 28]. VDD is clinically associated with an increased risk of type 2 diabetes mellitus, major cardiovascular events, cancers, infectious diseases, and chronic kidney disease [18]. VDD is also recognized as a marker of environmental and social conditions, influenced by factors such as latitude and seasonality, atmospheric pollution, gendered clothing practices, dietary resources, occupation, housing, and population density [23].

Vitamin D is a hormone precursor produced primarily by the action of type B ultraviolet light (UVB) on sterols in the skin [25]. Following successive activation steps in the liver and kidney, vitamin D hormone (calcitriol), works to promote the absorption of calcium and phosphorus from the intestine and reabsorption of calcium from the kidney for use by bone-producing osteoblasts [25]. Severe and prolonged deficiency of vitamin D, either through inhibition of cutaneous synthesis or poor dietary intake, is a leading cause of the metabolic bone diseases rickets and osteomalacia [26, 29]. These conditions are characterized by skeletal abnormalities occurring during endochondral growth and bone maintenance, respectively, although rickets is often used as an umbrella term for all the skeletal defects of VDD in children [26]. Here we use the term “rickets” to refer to the skeletal defects of VDD in children, “residual rickets” to refer to bone deformities of rickets that persist into adulthood, and “osteomalacia” to refer to the skeletal lesions of VDD experienced after growth is completed. The most characteristic of the gross lesions of VDD are deformities of the limbs and axial skeleton caused by muscular action and/or compressive load on soft, poorly mineralized bone [26, 30]. Macroscopically recognizable skeletal changes of VDD only occur after a prolonged period of deficiency or when the deficiency coincides with a period of rapid growth [29, 31]. It is likely that poor vitamin D status associated with shorter seasonal periods of deficiency and/or during periods of slower growth will be missed through macroscopic paleopathological analysis alone, because processes that would have occurred at the microstructural level prior to manifesting externally are not accessed and evaluated. Additionally, diagnostic lesions of childhood rickets may completely remodel in individuals who survived to adulthood, and the fragility of bone affected by this disorder means that it is less likely to survive in the post-depositional environment [30]. However, the clinical body of literature on vitamin D indicates that these archaeologically invisible periods of VDD had significant implications for population health, contributing to mortality and the burden of communicable and non-communicable diseases in the past [22].

### Sex distribution of vitamin D deficiency

Female sex has been found to be a clinical risk factor for VDD in both adults and children in several studies, although the reasons for this unclear [32–35], and the association is not universal [36]. Gendered social practices almost certainly play a role in some populations, with women in many cultures more likely to wear clothing that limits exposure to sunlight and to take part in indoor activities [23, 37]. However, an association with either sex has not been demonstrated in archaeological assemblages. Mays and colleagues used multivariate analysis to explore the relationship between latitude, sex, age, and settlement type (urban vs. rural) in a large cohort (N = 2,787) from Roman Europe [38]. The only variable they found to be significantly associated with VDD was northern latitude and only in the non-adult portion of the cohort [38]. This lack of association between sex and VDD here is unsurprising; VDD is notoriously difficult to recognize in adult skeletal remains and, until very recently, reliable methods of sex determination in subadult skeletal remains did not exist [39].

Traditional macroscopic sex estimation methods in bioarcheology are reliant on the presence and analysis of sexually dimorphic skeletal elements, including the pelvis and skull [40]. However, in cases where sexually dimorphic skeletal elements are absent, populations have less sexual dimorphism, or individuals are non-adults, macroscopic sex estimation methods cannot be used [41, 42]. In recent years, biochemical techniques have provided alternative methods for estimation of sex when macroscopic methods are not applicable [43–50], including in non-adults [39]. In this study, the sex of non-adults and adults of indeterminate sex is determined using the method of Stewart et al. [46, 47], which identifies X- and Y- chromosome linked dimorphic peptides of the amelogenin protein (AMELX and AMELY) recovered from tooth enamel using mass spectrometry. The method has been successfully applied to both modern and archaeological permanent and deciduous teeth, including teeth with minimal mineralization [44].

In the context of Industrial England, the presence of sex-related patterns in VDD in children is not currently known. This was a period in which for the first-time substantial numbers of women and girls were engaged in labor outside of the domestic sphere [20]. The Northeast of England was an epicenter for rickets during this period, but whether there was any sex-related pattern in prevalence has yet to be investigated.

### Vitamin D deficiency and dentinal development

Recently, histological techniques have provided an avenue for exploring vitamin D status in archaeological human remains, potentially allowing the detection of periods of poor vitamin D nutriture too brief or mild to result in macroscopic skeletal changes [51, 52]. The mineralization of all hard tissues, including tooth enamel and dentin, is dependent on sufficient vitamin D, calcium, and phosphorus within the body [53]. The organic matrix of dentin (predentin) is secreted by odontoblasts. These cells work along canaliculi-like dentin tubules, where predentin is gradually mineralized via the deposition of hydroxyapatite, a calcium-phosphate crystalline compound [54]. Interglobular dentin (IGD) describes a portion of the inner tissue of the tooth containing voids where zones of mineralization (calcospherites) in peritubular dentin, the hard tissue immediately surrounding dentin tubules, have failed to coalesce during development [54]. These defects are easily visualized in ground sections with light microscopy and appear as dark, scallop-shaped clusters within normal intertubular dentin, the more collagen-rich tissue found between tubules [51, 54]. Clinical research has found that the presence of IGD in humans is strongly associated with disorders of mineral metabolism suffered during infancy and childhood, particularly those related to defective vitamin D metabolism [55–57]. Studies examining archaeological human teeth have demonstrated that IGD occurs in individuals who also exhibit macroscopic skeletal lesions indicating rickets or osteomalacia [51, 58, 59]. Furthermore, due to the incremental nature of dentin formation, IGD episodes can be placed within specific chronological windows, allowing the potential identification of discrete periods of poor vitamin D status during development [60]. It is important to note, however, that an evidence-based threshold for what constitutes pathological IGD attributable to VDD has not yet been established and this is discussed further in section 4.2. Additionally, it is not known whether there may be sex-related differences in dentinal mineralization and, by extension, predisposal to IGD, although work with animal models has not found a relationship [61, 62].

Histological analysis of IGD has been used to explore vitamin D status in archaeological assemblages from the post-medieval Netherlands [59], medieval Wales [63], and medieval France [51]. However, previous studies of VDD in English archaeological assemblages have relied predominantly on macroscopic detection of skeletal rickets and osteomalacia (c.f. [64]). In this study, we aim to explore vitamin D status at Coach Lane in greater depth. We do this by assessing histological evidence of IGD and molecular evidence of chromosomal sex in a sub-sample of individuals with and without macroscopic evidence of rickets or residual rickets, to address the following research questions:

1. Macroscopic diagnosis of disease in dry bone has an inherent element of subjectivity and the lesions of VDD, particularly healed rickets and residual deformities of childhood rickets in adults, can be subtle. Do individuals at Coach Lane previously recorded as having gross anatomical changes attributable to childhood VDD also exhibit histological evidence of poor mineral metabolism during development in the form of IGD?
2. Seasonal VDD is a known environmental risk in the North of England [65]. Previous paleopathological work has found that the prevalence of rickets in the archaeological record increases with northern latitude [14, 16]. Is there evidence for periodic episodes of IGD in the teeth of individuals from Coach Lane that could be related to seasonal VDD?
3. Female sex has been identified as a risk factor for poor vitamin D status and seasonal VDD in some clinical cohorts [32, 34], although previous paleopathological research has not found evidence for this in adults [38]. Methods for biochemically assessing the sex of non-adults from archaeological remains have only recently been established [43]. Is there a statistically significant association of IGD presence with sex in the individuals at Coach Lane?

## Materials and methods

### Coach Lane, North Shields

The Coach Lane skeletal collection consists of 236 individuals. The site is located in North Shields, which lies within the borough of North Tyneside, to the east of Newcastle upon Tyne, and to the north of the River Tyne [66]. Excavations occurring in response to residential development encompassed the entire area of the cemetery, therefore all burials within this site were exhumed in 2010 [66].

In the early 19th century, North Shields was a populous port and market town, undergoing rapid development [67, 68]. The principal industries in the 18th and 19th centuries were fishing, shipbuilding, shipping of coal and lime, iron-foundries, salt works, and various manufacturing [67, 69]. Coach Lane was a former Society of Friends (Quaker) burial ground (AD 1711–1857), and the population interred in the cemetery was likely a mix of social statuses [66].

The skeletal remains of all individuals included in this study are held in the Department of Archaeology at Durham University in the United Kingdom. Identification numbers for each individual sampled are presented in Table 1. Permissions to carry out this study was issued by collection’s curators. No permits were required for the described study, which complied with all relevant regulations. Twenty-five adult and non-adult individuals were selected for histological analysis of IGD based on previously estimated age and sex. Regarding pathological lesions, the selection of individuals for the present study was blind whereby previously recorded diagnoses were not known when assembling the sub-sample. The estimated age-at-death and sex recorded previously were verified, and a second gross anatomical analysis of skeletal lesions was conducted prior to sampling for dental histology. Details on the individuals studied and the teeth sampled can be found in Table 1.

**Table 1:** Results of IGD analysis of sampled individuals.

Individual	Age-at-death (years)	Osteologically assessed sex	Teeth sampled	Number of IGD episodes	Age of IGD episodes	Macroscopic evidence of VDD
CL 21	1.5	U	dec mand m1	NA—heavy diagenesis	NA	yes
CL 118	3 to 4	U	dec mand m1, perm mand M1	2	birth, 6 months	yes
CL 25	3.5–4.5	U	dec max m1, perm max M1	0	NA	no
CL 14	3 to 5	F*	dec mand m1	0	NA	yes
CL 152	5 to 7	U	dec max m1, perm max M1	NA—heavy diagenesis	NA	no
CL 13	7 to 9	U	dec max m2, perm max M1	1	6–11 months	no
CL 57	9 to 11	M*	dec max m1, perm mand M1	2	0–6 months; 2.5 years	no
CL 84	10 to 12	U	dec mand m1, perm mand M1	1	2.5 years	no
CL 127	13 to 15	U	perm max M1	3	1, 2, 2.5 years	no
CL 107	14 to 16	M*	mand PM2	1	6 months	yes
CL 122	15 to 17	U	perm mand C	2	1, 2.5–5.5 years	yes
CL 87	15–20	M	perm max M1	2	0–6 months, 1–1.5 years	no
CL 66	20–34	F	perm mand C	10	1, 2, 3, 4, 5, 6, 7, 8, 9, 10 years	yes
CL 82	20–34	M	perm max C	5	1, 2, 2.5, 3, 4, 5.5 years	yes
CL 92	20–34	F	perm max M1	0	NA	no
CL 121	20–34	M	perm mand C	NA—sample failed	NA	no
CL 167	20–34	U	perm mand C	NA—sample failed	NA	yes
CL 247	20–34	M	perm max M1	1	birth-6 months	no
CL 63	35–49	F	perm mand M1	2	birth, 2.5 years	no
CL 123	35–49	M	mand PM1, max M3	5	2.5, 3.5, 4.5, 5–9, 12.5–16.5 years	no
CL 129	35–49	F	perm max C	4	1–1.5, 2–2.5, 3–4.5, 5.5	no
CL 234	35–49	F	perm mand M1	0	NA	yes
CL 258	35–49	M	perm mand C	2	3.5, 4.5	no
CL 120	50+	M	perm mand C	1	2 years	yes
CL 253	20+	F	perm mandibular C	0	NA	no

U = unknown, F = female, M = male, dec = deciduous, perm = permanent, mand = mandibular, max = maxillary, m/M = molar, C = canine, PM = premolar, NA = not applicable.

*chromosomal sex previously determined [39]

### Macroscopic analyses

Paleopathological analysis of metabolic bone disease and age-at-death estimation for the non-adults from this site were previously conducted by SN, RG and AC and published elsewhere [16]. All adults analyzed in the present study were macroscopically examined by AS under natural light for abnormalities of bony proliferation, destruction, size, and shape following discipline standards [70]. Differential diagnosis of active rickets, healed rickets, residual rickets, and osteomalacia was conducted after Brickley et al. [30, 71, 72]. Because the lesions of residual rickets can be subtle and overlap with several other conditions, Brickley and colleagues advocate a highly conservative approach to differential diagnosis [73]. For the purposes of this study, we have included any adult individual with evidence of bowing deformity of the limbs recorded independently by two of the authors as a possible case of residual rickets, recognizing that this may overestimate the prevalence of this condition. Adult age estimation was conducted following the standards outlined in Buikstra and Ubelaker [40, 74, 75]. Adults were placed into broad age-at-death categories of young (20–34 years), middle (35–49 years), and older (50+ years) to account for the high degree of error associated with these methods. Adult sex was estimated via cranial and pelvic sexually dimorphic features as per methods contained in Buikstra and Ubelaker [40] and Bruzek [76]. Three non-adults from the sample, CL 14, CL 57, and CL 107, had previously had their sex established by enamel peptide analysis [39].

### Histological analysis

At least one tooth was selected from each of the 25 individuals for histological analysis. Exceptions were subadults with mixed dentition where one permanent and one deciduous tooth were sampled to provide a longer chronological record for the presence of dentinal defects. First permanent molars were preferentially selected as these provide a record of the late *in-utero* period through the first 10 years of postnatal development [60, 77]. Where first molars were unavailable, permanent canines were selected as these provide a dentinal record of the first 12–14 years of postnatal life [60, 77]. In one case (COL 123), a first mandibular premolar was selected in addition to the right maxillary third molar as these provide a record of the first ~1.5–12 years of development and no other teeth were available to sample. In another case (CL 107) only a second mandibular premolar, providing a record of ~2.5–13 years of development, was available to sample. In total, 22 molars (12 upper, 10 lower), 8 canines (2 upper, 6 lower), and the 2 lower premolars were sampled from 25 individuals. Twenty-four teeth were from the right side and 8 were from the left side. Prior to sectioning, all teeth were photographed, first *in-situ* and then following extraction (if applicable) and radiographed on a Carestream Point-of-Care digital CR reader radiography machine (60kVp and 0.640mAS).

Because the teeth studied here were designated for several types of destructive analyses, including future isotopic work, they were manually sectioned longitudinally into two portions. Using a dental drill equipped with a diamond cutting blade, the teeth were cut as close as possible to the tooth’s central axis in either a mesio-distal (molars) or labio-lingual (anterior teeth) direction. One portion of the tooth was retained for future work. Given the imprecise nature of manual sectioning, there was some inconsistency in the size of tooth portions. However, this should not have a major impact on our recording of IGD; as this is a qualitative study, we are not recording enamel or dentin increments quantitatively, and a consistent location of a histology slice was further cut on a low-speed saw. The portions for histological analysis were made into thin sections following standard methods [78]. Each sample was embedded in Buehler EpoThin2™ epoxy-resin. A Buehler IsoMet™ high speed saw equipped with diamond wafer blade was used to section the embedded blocks longitudinally through each tooth sample in a mesio-distal/labio-lingual plane. The ‘exposed’ surface of the tooth was briefly smoothened with a 600grit/P1200 silicon carbide paper and mounted on a glass slide with Araldite™ adhesive. Final reduction to a thickness of ~100–120μm was undertaken with successively finer grits (320grit/P400, 600grit/P1200) of silicon carbide paper on a Buehler EcoMet™ manual grinder-polisher. The sample was then polished with Buehler MicroPolish II 0.3μm powder, sonicated to remove debris, dehydrated with 90% and 100% ethanol, and cleared with xylene. Imaging was conducted on an EVOS M5000 microscope under transmitted light and sections were visually inspected at 40x, 100x, and 200x total magnification. Scanned images of each section were stitched in Adobe Photoshop (v. 6) to create complete 40x virtual slides and 100x regions of interest (ROIs) of any dentin containing IGD voids. Image analysis was conducted in FIJI™ software and IGD was assessed using the Snoddy et al. gray-scale histogram protocol [52], which estimates the cumulative value of IGD voids present in a 0.5 x 0.5mm area of dentin. The D’Ortenzio et al. grading system [51] of IGD severity was also employed as a supplementary method as some samples contained heavy diagenesis or staining that made it impossible to visually isolate IGD voids via image thresholding. Developmental IGD (DIGD) was defined conservatively after D’Ortenzio and colleagues as widely spaced, scattered voids within the mantle dentin that lack an incremental banding pattern and fall under grade 1 [79]. The timing of IGD episodes was estimated according to Brickley et al. [60] and AlQahtani et al. [77]. The timing of IGD formation was assessed to identify discrete episodes of VDD during development and determine their frequency and periodicity.

### Enamel peptide analysis

Enamel peptide samples were collected from eight non-adult individuals aged from 18 months to 17 years old, and from two adult individuals whose sex could not be estimated macroscopically. Details of the teeth sampled for peptide analysis can be found in S1 Table.

The samples were collected at Durham University and subsequently analyzed at the University of Brighton. The nanoLC-MS/MS methods used in this study are based on the methods described in Stewart et al. [46, 47], which uses a minimally destructive acid-etching procedure. A full description of the methods for enamel peptide analysis can be found in S1 File.

The datasets generated during and/or analyzed during the current study are available in the ProteomeXchange Consortium (http://www.proteomexchange.org/) via the PRIDE [1] partner repository with the dataset identifier PXD038154.

### Statistical analysis

The association between sex and IGD presence and sex and macroscopic evidence of VDD was explored using a Fisher exact test (Vassarstats). The significance threshold (α) was assigned as ≤0.05.

## Results

Thin sections from CL 21 and CL 152 did not yield any recoverable histological data due to heavy diagenesis (likely of fungal origin), and thin sections from CL 167 and CL 121 failed due to technical issues. These samples were excluded from this study. We report results for a total of 21 individuals with thin sections of suitable quality for IGD examination. Of the 21 individuals, 16 (76.1%) exhibited at least one clear episode of IGD. Nine individuals (42.8%) exhibited more than one IGD episode and four individuals, (19%) exhibited four or more episodes at regular intervals. One individual, CL 234, exhibited a few scattered IGD voids along the dentin-enamel junction but was discounted as a possible pathological case since these likely represent a developmental artifact [79]. The severity of IGD episodes observed was highly variable according to both the Snoddy et al. [52] and D’Ortenzio et al. [51] protocols, ranging from 2.3% per 0.5mm^2^ / grade I to 58% per 0.5mm^2^ / grade III between individuals. Results are summarized in Table 1 and outlined below. Details on the scoring of IGD episodes can be found in S2 Table.

### Comparison to macroscopic data

**Fig 1:**
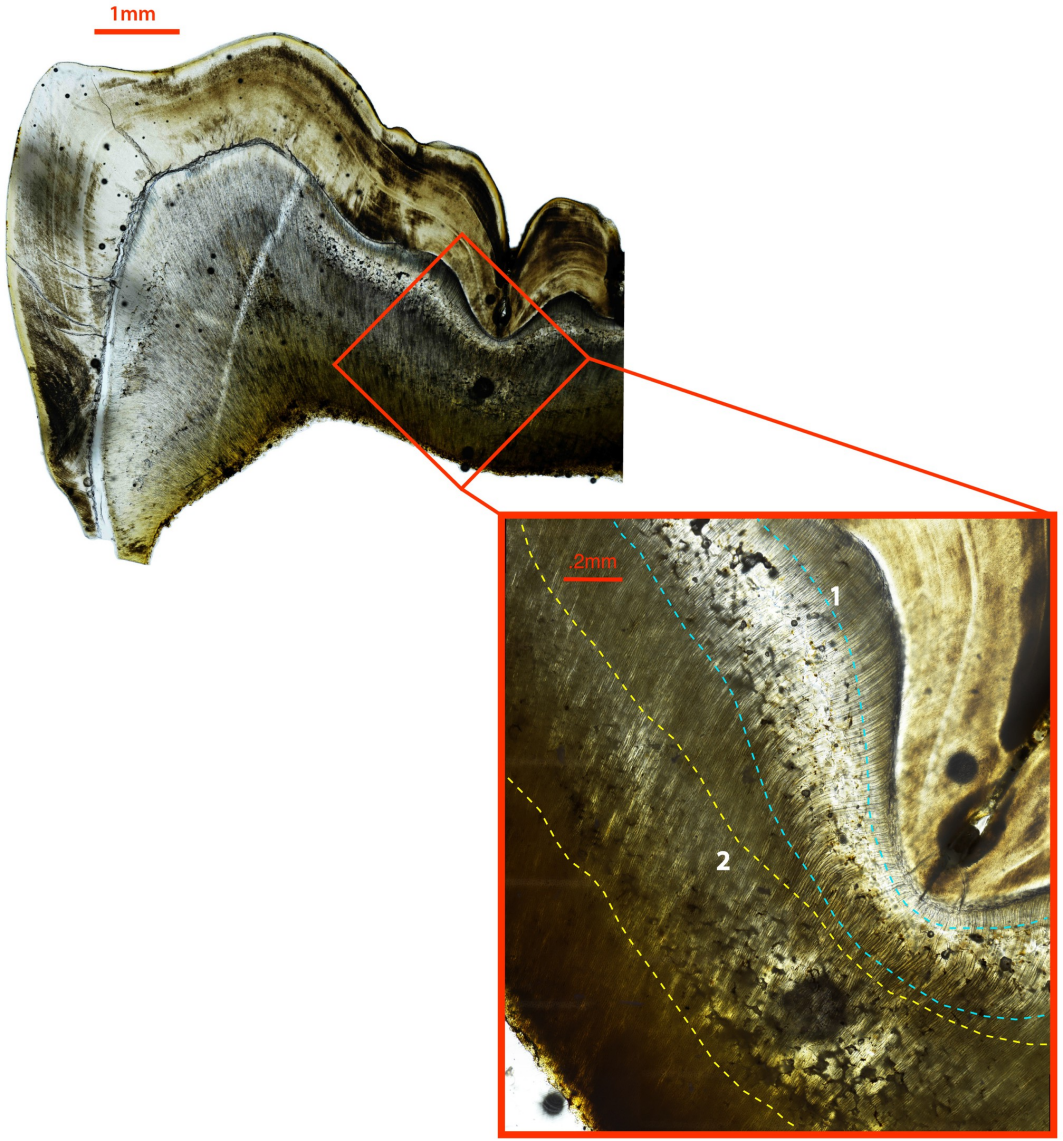
Ground section of the right permanent mandibular first molar of CL 118. Two bands of IGD are visible, the first occurring ~birth-6 months (teal dashed lines) and the second occurring between ~1 and 1.5 years (yellow dashed lines). Large image is 40x total magnification, inset box is 100x total magnification.

Of the 16 individuals with clear evidence of IGD, only six (37.5%), three subadults and three adults, exhibited macroscopic evidence of VDD in the form of active, healed, or deformities due to possible residual rickets (S3 Table). We discuss each of these cases here. CL 107, a 14-16-year-old male adolescent, exhibited slight bowing of the long bones, flared metaphyses, costochondral beading, and tibial torsion. This individual had one episode of IGD in their left permanent mandibular first molar, occurring at ~6 months of age. CL 118, a female child between 3 and 4 years old, exhibited evidence of possible rickets in the form of bowing deformities and heavy metaphyseal porosity in the left and right femora, tibiae, and fibulae. Two clear bands of interglobular dentin were present in the partially formed crown of the right permanent mandibular molar (Fig 1). The first of these occurred between birth and ~6 months and the second between 1 and 1.5 years. CL 120, an older adult male, had been previously recorded as exhibiting bowing deformities in their lower limbs, including *genu valgum* [21]. This individual had one distinct episode of IGD in their right permanent mandibular canine, occurring at ~2 years of age. CL 122, a female adolescent exhibited bowing of the femora, and heavy porosity in the femoral neck, mandible, and anterior vertebral bodies, suggestive of some form of metabolic bone disease. They exhibited two episodes of IGD in the crown of their right permanent mandibular canine. The first of these occurred around 1 year and the second appears to have formed continuously between ~2.5–5.5 years. Finally, CL 66 and CL 82, discussed in more detail below, exhibited evidence of residual rickets in the form of long bone bowing and both also exhibited multiple episodes of IGD throughout childhood.

### Evidence of cyclic IGD episodes

**Fig 2:**
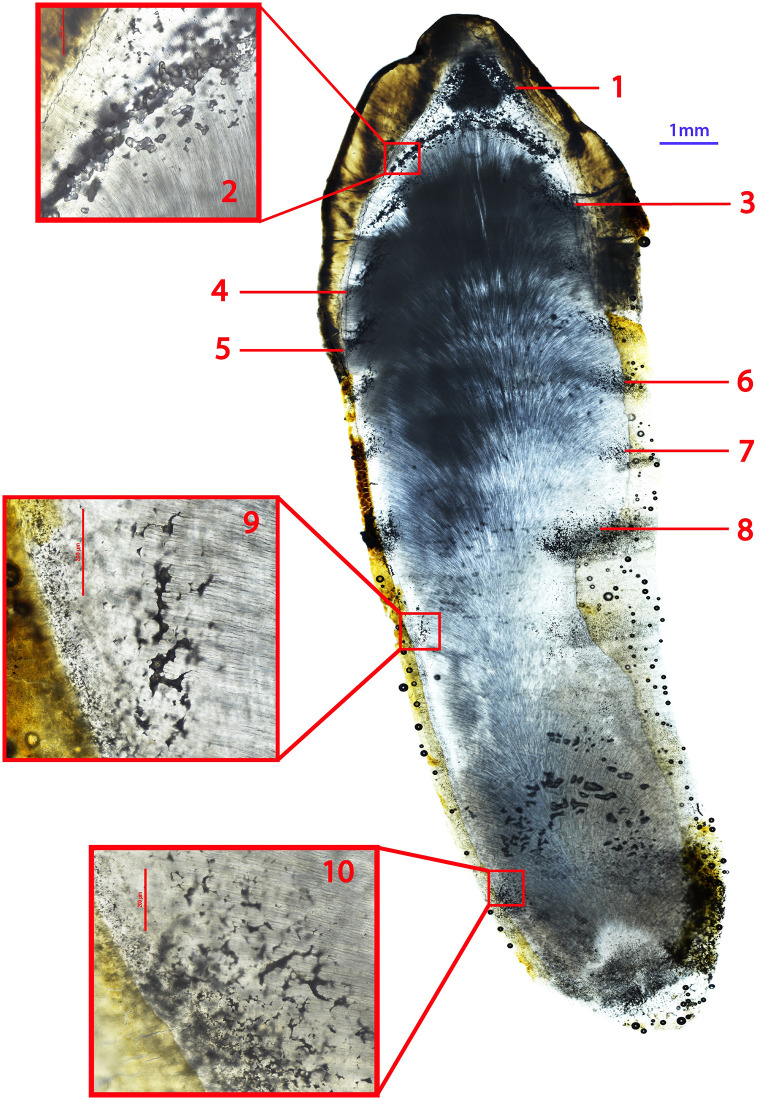
Ground section of the right permanent mandibular first molar of CL 66. Ten episodes of IGD between crown and apex are visible. Whole tooth is 40x total magnification, inset boxes are 100x total magnification. Note that because this is a very lateral section, episodes 7–10 are partially obscured by the overlying cementum and Granular Layer of Tomes.

**Fig 3:**
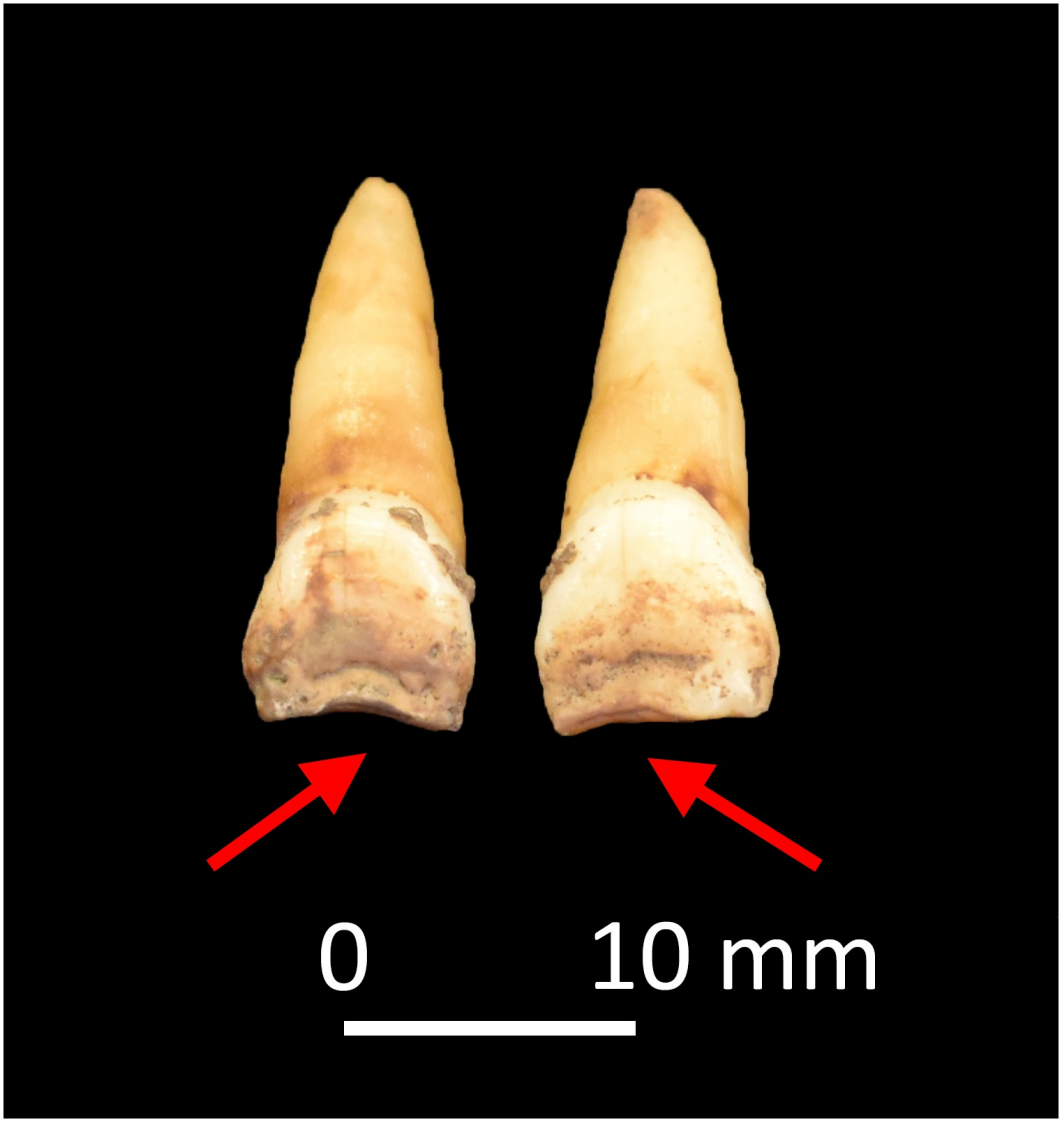
Permanent central maxillary incisors of CL 66. Plane form hypoplasia is exhibited on the occlusal and labial surfaces.

Four adults, CL 66, CL 82, CL 123, and CL 129, two of whom had skeletal evidence suggestive of residual rickets, exhibited four or more discrete IGD episodes occurring at regular intervals (Table 1). These are generally equidistant from each other and appear to occur in approximately annual increments according to Brickley and colleagues’ dentin formation chart [60]. CL 66, a young adult female, exhibits 10 episodes in their right permanent mandibular canine, beginning at the tip of the coronal dentin and extending down the root (~1–13 years) (Fig 2). In addition to macroscopic bowing of the tibiae, this individual also exhibited notching of the crowns of their permanent central maxillary incisors (Fig 3), a form of enamel hypoplasia associated with both congenital syphilis and vitamin D deficiency in infancy [80, 81]. CL 82, a young adult male, exhibited macroscopic evidence suggestive of residual rickets in the form of bowing of the right humerus and femur. This individual had five episodes of IGD, beginning at one year of age and recurring approximately every year until 5.5 years of age (Table 1). The regular nature of the IGD episodes in these four individuals and the fact that they appear to occur annually suggests seasonal changes in mineral metabolism. The implications of this are discussed below.

### Sex distribution of individuals with IGD

**Fig 4:**
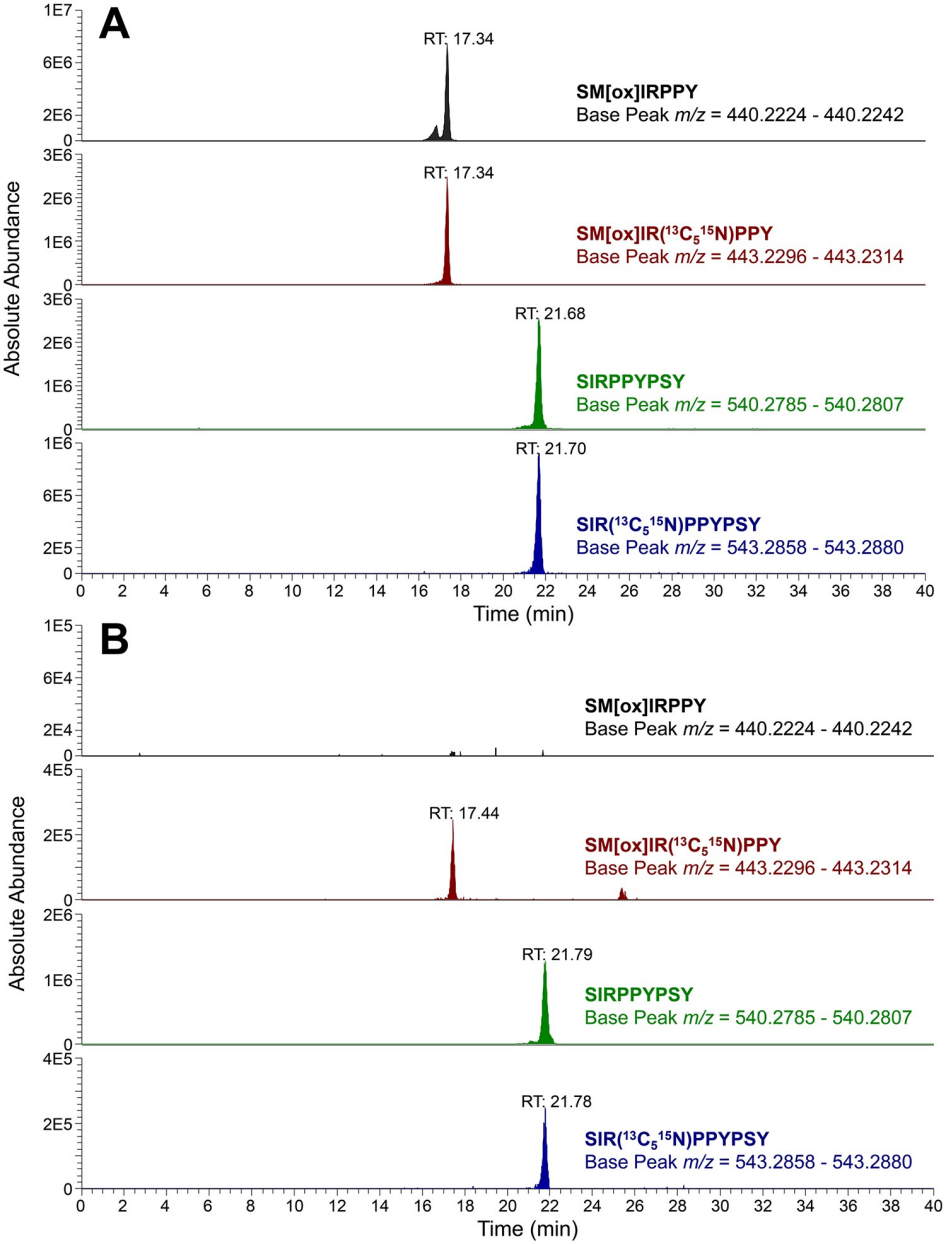
Enamel peptide results for CL 152 (XY, male)(A) and for CL 127 (XX, female)(B).

Of the 10 individuals sampled for peptide analysis, three were identified as chromosomally male (SM[ox]IRPPY and SIRPPYPSY were identified), and six were identified as chromosomally female (only SIRPPYPSY was identified) based on the ion chromatography results. One individual (CL 122) was identified as a probable female due to only a very small amount of endogenous SIRPPYPSY being present in the sample (although no endogenous SM[ox]IRPPY and SIRPPYPSY were identified in the sample either, lending strength to the possibility of this individual being female). The chromosomal sex of the 10 sampled individuals is included in Table 2. Reconstructed ion chromotagrams for CL 152 (male) and CL 127 (female) are shown in Fig 4.

**Table 2:** Sex estimation results from the peptide analysis of eight non-adults and two adults.

Individual	Tooth Sampled	Peptide Results
CL 13	Left max M1	M
CL 21	Left max M1	F
CL 25	Right max M1	F
CL 84	Right max m2	M
CL 118	Left mand m1	F
CL 122	Left max M2	F*
CL 127	Right max M2	F
CL 152	Left max PM1	M
CL 167	Left mand PM1	F
CL 253	Right max I2	F

*likely female; very few peptides recovered from the enamel matrix. dec = deciduous, perm = permanent, mand = mandibular, max = maxillary, m/M = molar, C = canine, PM = premolar, I = incisor

Overall, 54.5 percent of females (6/11) with analyzable ground sections exhibited at least one episode of IGD vs 100% (10/10) of males. The presence of IGD was significantly associated with male sex by a Fisher’s exact test (p = .0351). All males with macroscopic evidence of VDD also exhibited at least one episode of IGD. More females exhibited macroscopic evidence of VDD (45.4% vs 30%), but this was not statistically significant (p = .6594). An equal number of males and females (two and two) exhibited evidence of cyclic/periodic IGD.

## Discussion

### Vitamin D status at North Shields: Integrating lines of evidence

The overwhelming majority of individuals in our histological sample (76%) had at least one episode of IGD. Tschinkel and Gowland [21] reported that 18% (26/143) of adults from this site had macroscopic evidence of VDD or residual deformities attributable to childhood rickets while Newman et al. [16] noted that 25.9% of the non-adults at Coach Lane exhibited macroscopic evidence of rickets. If it is accepted that all 16 individuals with IGD reported here suffered from poor vitamin D status, it appears that this was a much wider problem than the already high macroscopic prevalence of rickets and residual rickets suggests. Periods of poor mineral metabolism during dentinal development are practically ubiquitous in our sample. Given what is known of the environmental and social context of this site, this may be attributable to VDD or VDI experienced during growth.

The apparent seasonality of these episodes in several individuals, as evidenced by annual IGD episode periodicity, further supports the hypothesis of poor vitamin D status as an etiology for these defects. Although inborn errors of vitamin D metabolism and even normal dentinal development can also produce interglobular defects, these are not cyclic [54, 57]. Similar histological findings of probable seasonal IGD have been reported in two post-medieval Dutch assemblages [59]. This also suggests that VDD at North Shields has a significant environmental component related to latitude. If the high prevalence of VDD at this site was primarily attributable to the effects of industrialization–that is, low UVB exposure due to long factory days and crowded, terraced housing–the periodicity between episodes would be less regular. Historical records also attest to a seasonal component in rickets prevalence in England with more cases reported in the spring [82], presumably representing the physical consequences of autumn and winter VDD. Our findings are consistent with the work of Newman and colleagues who found a significantly higher prevalence of macroscopic indicators of VDD in children from Northern English archaeological assemblages [16], as well as with the clinical literature from this region [65]. Intriguingly, isotopic analysis of this assemblage indicates that despite the coastal proximity of this site, this population consumed very little marine fish, an important dietary source of vitamin D that may have acted as a buffer to seasonal deficiency [83]. There can be little doubt, given the cultural context, that social factors, including infant feeding practices also played a role in the prevalence of VDD in the Coach Lane assemblage [16, 84]. Early weaning or even full artificial infant feeding were common during the industrial period [85] and this likely contributed to disorders of mineral metabolism. Weaning onto contaminated foods increases the risk of developing rickets due to poor calcium absorption from gastrointestinal illness, whilst giving cow’s milk as a substitute disrupts infant calcium metabolism [6]. This highlights the etiological complexity of poor vitamin D status and the need to consider environmental and social context in tandem. Future work might explore the periodicity of IGD in contemporaneous industrial assemblages from more southern latitudes.

As discussed in section 1, poor vitamin D status is clinically associated with several negative health outcomes unrelated to bone health, including increased risk for infectious diseases, cardiovascular disease, cancers, and all-cause mortality [22]. Most of these conditions do not leave evidence in skeletal remains, but it would be remiss not to consider the wider implications of poor vitamin D nutriture on morbidity, mortality, and quality of life in industrial North Shields. In their analysis of non-adults from this site, Newman and colleagues found that the assemblage exhibited a higher co-occurrence of metabolic disease with other ‘stress’ indicators (cribra orbitalia and dental enamel hypoplasia), and high rates of pathology in general, when compared to the pooled southern English archaeological sample [16]. They postulate that this may reflect the negative impact of poor vitamin D status on extraskeletal processes, such as immunocompetence. Our histological results bolster this interpretation by providing some potential evidence for more widespread poor vitamin D nutriture than indicated by macroscopic analysis of skeletal remains.

### The pathophysiology of IGD and implications for assessing vitamin D status

Some of the individuals from Coach Lane with more severe and prolonged histological evidence for poor mineral metabolism did not exhibit any macroscopic lesions attributable to this VDD. For the adults this is not unexpected, as they may have had sufficient time to completely remodel skeletal lesions before their death [30]. However, CL 13, CL 57, and CL 84, all non-adults aged 7–12 years, exhibited one or more episodes of IGD and no macroscopic evidence of rickets other than some slight metaphyseal porosity in CL 13. It is possible that any macroscopic lesions in the bones of these children had remodeled, particularly as the last episode of IGD exhibited by any of them was at 2.5 years. If the physiologically intuitive idea that IGD begins to form as soon as the circulating pool of vitamin D drops to a level at which mineralization of predentin cannot occur is accepted [51], it follows that these individuals could also represent the macroscopically “invisible” portion of the population of North Shields affected by poor vitamin D status. Because dentin forms incrementally and does not remodel, periods of poor mineral metabolism too brief to result in clinical rickets can theoretically be retrospectively identified through analysis of IGD alone. Analogous changes are expected to simultaneously occur in bone in the form of an accumulation of unmineralized osteoid [86, 87]. However, because bone is continuously remodeling throughout life, histological analysis of this tissue will only detect active or very recent cases of VDD.

CL 14, a child aged between 3–5 years who exhibited evidence of healed rickets in the form of lower limb bowing, did not exhibit any IGD in the section taken from her left mandibular deciduous first molar. This tooth should provide a record of dentinal development from ~birth through to 3 years old [60]. We do note that this tooth was affected by possible fungal diagenesis in the distal 2/3rds of dentin, so it is possible that an episode or episodes of IGD were missed. However, it is also possible that the apparent bowing exhibited in this person was due to another condition, or normal anatomical variation [30]. This shows that histological analysis of IGD may have the potential to “ground truth” macroscopically diagnosed cases of VDD, particularly individuals purported to have residual rickets based on skeletal lesions alone. However, an appropriate dentinal chronology would need to be sampled.

It is important to note that the explicit relationship between IGD severity (generally accepted to be the relative proportion of intertubular dentin containing interglobular voids [51, 52, 88–90]), IGD episode duration, and clinical measures of VDD severity (i.e. serum pro-hormone levels, radiological changes) has not been established. Although scoring systems for IGD in anthropological tooth sections have been created [51, 52, 88, 89], it is not yet known if more “severe” IGD translates to objectively worse vitamin D status in the individual and there does not appear to be a consistent relationship between the severity of macroscopic lesions of VDD and higher histological IGD scores. Indeed, the presence of IGD appears to be linked to some degree to growth of the tooth itself, and its distribution varies between the tooth root and crown [90]. Until an evidence-based pathological threshold for IGD is established, caution is needed in using its presence in archaeological thin sections as a proxy for clinical rickets. Bearing the above caveat in mind, our findings highlight the utility of histological methods as a complement to macroscopic ones. They also have the potential to provide a more objective measure of poor childhood vitamin D status than macroscopic rachitic lesions like limb bowing, which can be subtle and temporary.

**Fig 5:**
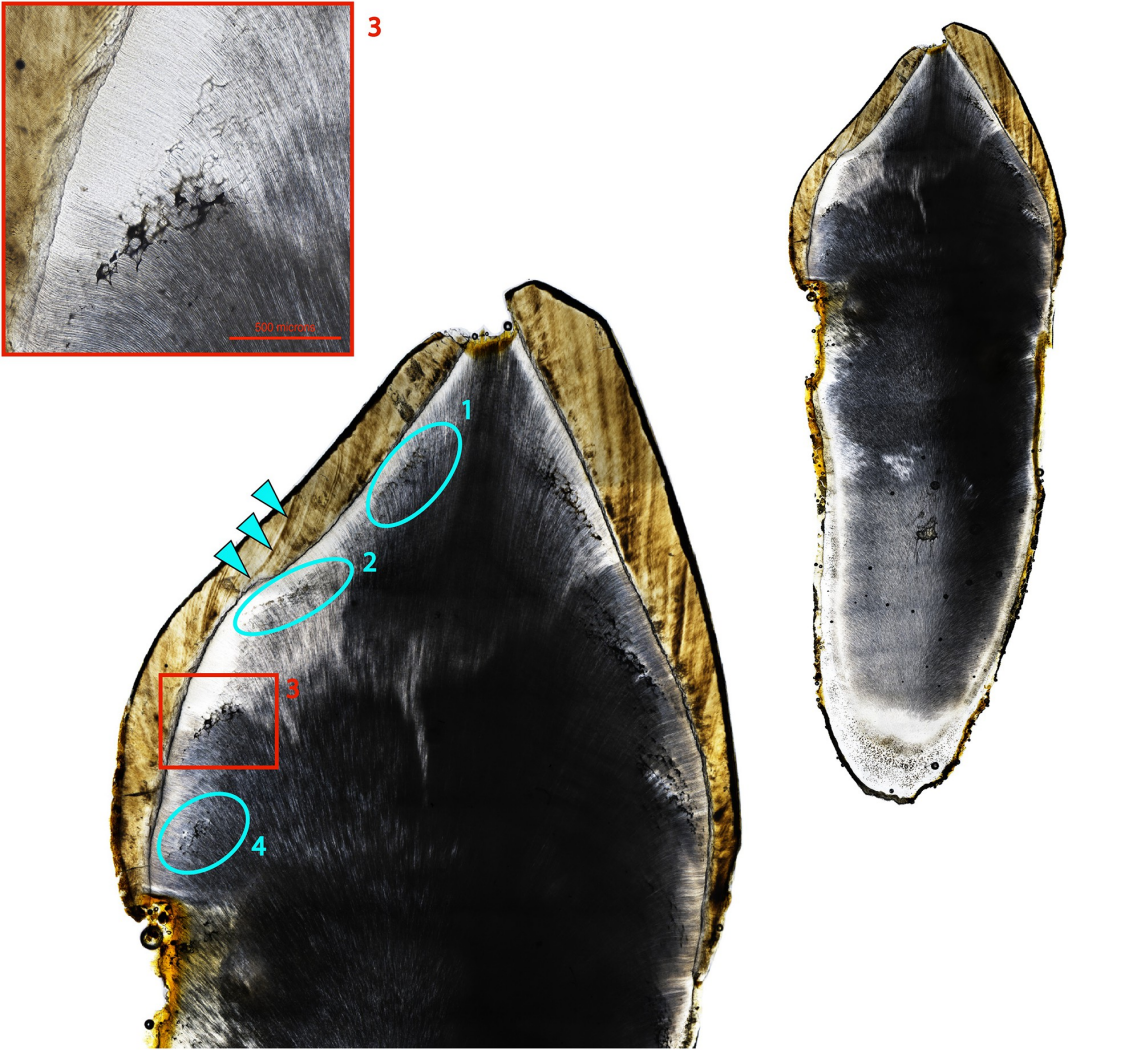
Right permanent maxillary canine of CL 129. Four discrete episodes of IGD in approximately annual increments are apparent. Teal arrows indicate a Wilson band formed concurrently with the 2nd episode of IGD. Whole tooth and crown images are 40x total magnification (not to scale), inset box is 100x total magnification.

An incidental finding of our histological analysis of dentin was the concurrent formation of Wilson’s bands in the enamel of several individuals (Fig 5; S2 Table). These accentuated striae of Retzius represent developmental periods of abnormally slowed amelogenesis [54]. The presence of Wilson bands in chronological association with episodic IGD bolsters the likelihood that these represent discrete periods of poor systemic mineral metabolism and are not just an artifact of dentinal development. However, we could not systematically test this relationship as the posterior teeth in our sample were sectioned in a meso-distal direction and analysis of enamel secretion requires examination of a consistent bucco-lingual/labio-lingual section of each tooth [78, 91]. Given that the samples used in this study were manually portioned residuals, any thin sections of anterior teeth that were cut in a labio-lingual plane were not of suitable quality for high resolution analysis of enamel growth increments. They were further compromised by post-depositional staining obscuring microscopic expression of enamel and dentin increments. Future work might explore the association of accentuated striae in enamel with IGD and use enamel secretion rates to estimate the time between, and duration of, IGD episodes in coronal dentin with high precision. This may also give more insight into the possible seasonality of episodes.

### Sex distribution of macroscopic rickets and IGD

The association between male sex and the presence of IGD is intriguing. If IGD is truly a marker of poor vitamin D status, this may indicate that males were at higher risk for VDD than females at Coach Lane. Veselka and colleagues [59] reported that adult males exhibited more “severe” episodes of IGD than females in their samples from post-medieval Holland, with females exhibiting visible IGD on thin sections but not by microCT scan, and they found no association between sex and IGD presence. Although our sample size is also small, we were able to demonstrate a statistical association between male sex and IGD presence. This could be attributable to type I error but proceeding on the assumption that this is a true association, there are two potential explanations. First, the male children at Coach Lane were truly at higher risk for poor vitamin D status. This is counter-intuitive given the biocultural context of industrial England where female children were more likely to cover their skin and take part in indoor activities. It could be argued that the rise in child labor during this period meant that male children were more likely to be part of the workforce from a young age [92]. Regional industries like coal mining would have been especially high risk for VDD. However, all the Coach Lane males, except for CL 123, exhibited evidence of IGD before 5 years of age which indicates that child labor practices are unlikely to be the underlying cause. CL 123 did exhibit evidence of IGD in their third maxillary molar which captures the adolescent period. However, they also exhibit regular episodes beginning at 2.5 years of age in their first mandibular premolar which means that labor practices are an unlikely cause, and Kirby [93] has suggested that poor health seen in children during this time likely stemmed from conditions within the home environment, prior to their entrance into the labor market. A large study of primary care records of UK children (N = 711,788) found that in the under 5-year category, males were more likely to be diagnosed with VDD whilst females were more likely to be diagnosed in the >10-year age category [36]. The authors suggest that modest dress of older female children in some communities may drive the higher rates of VDD in this group. Our results are consistent with these findings, and it appears that young male children at Coach Lane may truly have a higher risk of developing VDD, although the biological and social reasons for this need further exploration.

The second explanation for the association of IGD with male sex is related to sex-differences in dentinal mineralization itself. It could be that males are inherently more likely to develop IGD. This is a clinically underexplored topic and limited experimental animal work has not found an association between sex and dentin mineralization [61, 62]. This is an area where future research is needed, particularly given the increasing popularity of IGD studies in bioarchaeological work.

## Conclusion and suggestions for future research

Our dental histological analysis has contributed towards a better understanding of vitamin D status in industrial Northern England. IGD, indicative of periods of poor mineral metabolism, was widespread at Coach Lane with over 76% of individuals examined exhibiting one or more episodes. This is far higher than macroscopically recorded cases of rickets in this assemblage, which may suggest that the individuals with gross anatomical changes represent only a small proportion of a population suffering from poor vitamin D status during growth. Several individuals exhibited evidence of seasonal episodes, suggestive of an environmental deficit of vitamin D due to latitude. Although females were more likely to exhibit skeletal evidence of VDD, male sex was found to be statistically significantly associated with the presence of IGD. It is unclear if this is because males are at higher risk for poor vitamin D status or if this could be attributable to sex related differences in dentinal mineralization itself. An equal number of males and females exhibited cyclic annual episodes of IGD.

Future work might compare histological evidence of VDD in assemblages from more southern latitudes. Incorporation of amelogenin peptide analysis on larger samples from other assemblages would build on the work begun here, providing a more complete epidemiological picture of environmental and biosocial factors in the prevalence of this disease in the past. The relationship between accentuated striae in enamel and the presence of IGD in dentin is another avenue that merits exploration; this may be particularly useful in exploring IGD episode seasonality as accentuated striae can be chronologically placed with more accuracy than dentinal defects.

## Supporting information

S1 File Enamel peptide analysis protocol. (DOCX)

S1 Table Details of teeth sampled for peptide analysis. (DOCX)

S2 Table IGD episode scores by individual. (XLSX)

S3 Table Macroscopic skeletal evidence of VDD. (XLSX)
