# Correction: Vitamin D status in post-medieval Northern England: Insights from dental histology and enamel peptide analysis at Coach Lane, North Shields (AD 1711–1857)

**DOI:** 10.1371/journal.pone.0333161

**Published:** 2025-09-23

**Authors:** Anne Marie E. Snoddy, Heidi Shaw, Sophie Newman, Justyna J. Miszkiewicz, Nicolas A. Stewart, Tina Jakob, Hallie Buckley, Anwen Caffell, Rebecca Gowland

There is an error in the caption for Fig 2, “Ground section of the right permanent mandibular first molar of CL 66.” Please see the complete, correct Fig 2 caption here.

In Table 1, the sample for individual CL 107 should have been “perm mand M1.” Please see the correct Table 1 here.

**Table 1:** Results of IGD analysis of sampled individuals.

Individual	Age-at-death (years)	Osteologically assessed sex	Teeth sampled	Number of IGD episodes	Age of IGD episodes
CL 21	1.5	U	dec mand m1	NA – heavy diagenesis	NA
CL 118	3–4	U	dec mand m1, perm mand M1	2	birth, 6 months
CL 25	3.5–4.5	U	dec max m1, perm max M1	0	NA
CL 14	3–5	F*	dec mand m1	0	NA
CL 152	5–7	U	dec max m1, perm max M1	NA – heavy diagenesis	NA
CL 13	7–9	U	dec max m2, perm max M1	1	6-11 months
CL 57	9–11	M*	dec max m1, perm mand M1	2	0-6 months; 2.5 years
CL 84	10–12	U	dec mand m1, perm mand M1	1	2.5 years
CL 127	13–15	U	perm max M1	3	1, 2, 2.5 years
CL 107	14–16	M*	perm mand M1	1	6 months
CL 122	15–17	U	perm mand C	2	1, 2.5–5.5 years
CL 87	15–20	M	perm max M1	2	0−6 months, 1–1.5 years
CL 66	20–34	F	perm mand C	10	1, 2, 3, 4, 5, 6, 7, 8, 9, 12 years
CL 82	20–34	M	perm max C	5	1, 2, 2.5, 3, 4, 5.5 years
CL 92	20–34	F	perm max M1	0	NA
CL 121	20–34	M	perm mand C	NA – sample failed	NA
CL 167	20–34	U	perm mand C	NA – sample failed	NA
CL 247	20–34	M	perm max M1	1	birth-6 months
CL 63	35–49	F	perm mand M1	2	birth, 2.5 years
CL 123	35–49	M	mand PM1, max M3	5	2.5, 3.5, 4.5, 5–9, 12.5–16.5 years
CL 129	35–49	F	perm max C	4	1–1.5, 2-2.5, 3–4.5, 5.5
CL 234	35–49	F	perm mand M1	0	NA
CL 258	35–49	M	perm mand C	2	3.5, 4.5
CL 120	50+	M	perm mand C	1	2 years
CL 253	20+	F?	perm mandibular C	0	NA

U = unknown, F = female, M = male, dec = deciduous, perm = permanent, mand = mandibular, max = maxillary, m/M = molar, C = canine, PM = premolar, NA = not applicable.

*chromosomal sex previously determined [39]

**Fig 2:**
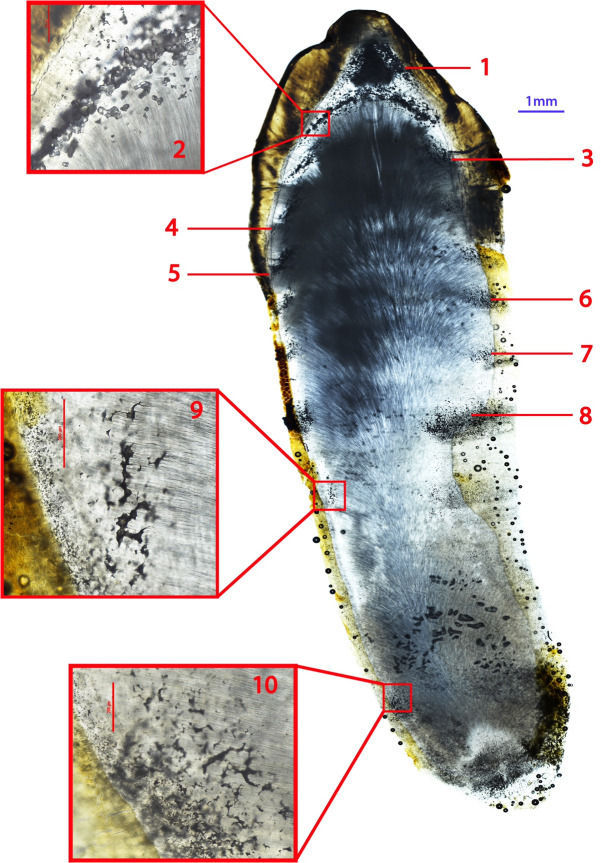
Ground section of the right permanent mandibular canine of CL 66. Ten episodes of IGD between crown and apex are visible. Whole tooth is 40x total magnification, inset boxes are 100x total magnification. Note that because this is a very lateral section, episodes 7–10 are partially obscured by the overlying cementum and Granular Layer of Tomes.
