# Supplementary material for: Vitamin D status in post-medieval Northern England: Insights from dental histology and enamel peptide analysis at Coach Lane, North Shields (AD 1711–1857)
Source: PLoS One. 2024 Jan 31;19(1):e0296203. doi: 10.1371/journal.pone.0296203 (PMC10830048; doi:10.1371/journal.pone.0296203)
Supplement: S1 File — (DOCX) [file pone.0296203.s001.docx]

Enamel peptide analysis

An initial etch was performed by releasing approximately 40 μL of 5% (vol/vol) HCl onto the tooth fragment and maintaining contact via surface tension for 2 mins; this first etch was retained for analysis. The process was repeated for a second time and the etch solution was again retained. A C18 resin loaded ZipTip (ZTC18S096; EMD Millipore) was conditioned three times with 100% acetonitrile using a 10 μL pipette, followed by three times with 0.1% (vol/vol) formic acid, before being discarded. The peptides were bound to the ZipTip by drawing the etch solution through the ZipTip 10 times, discarding the last draw. The ZipTip was then washed six times with 0.1% (vol/vol) formic acid; each wash was discarded. The resin-bound peptides were eluted by drawing a 4-μL 60% acetonitrile/0.1% formic acid elution buffer through the ZipTip 10 times and the eluted peptides subsequently lyophilized. Samples were dissolved in 10 μL of a solution containing the heavy labelled internal standard peptides (Ser-Met(oxidized)-Ile-Arg-(^13^C^15^N)Pro-Pro-Tyr and Ser-Ile-Arg-Pro-(^13^C^15^N)Pro-Tyr-Pro-Ser-Tyr, AQUA, Sigma) at a concentration of 5 pmol/mL in 0.1% trifluoroacetic acid in water and transferred to glass autosampler vials.

A sample of 5 μL of the first etch was injected for analysis by reversed-phase nanoLC–MS (UltiMate 3000 RSLCnano; Thermo Fisher Scientific) coupled to a hybrid quadrupole Orbitrap mass spectrometer (Q Exactive Orbitrap; Thermo Fisher Scientific) equipped with a nanospray ion source (Nanospray Flex, Thermo Fisher Scientific). Peptides were first loaded onto a trapping cartridge (Pepmap100 C18; Thermo Fisher Scientific; 0.3 × 5 mm i.d.; 5 μm particle size) a flow rate of 20 μL/min for 4 min using mobile phase A (0.1% [v/v] formic acid in hypergrade water, Merck KGaA). Peptides were eluted on an analytical column (PepMap100 C18; 15 cm × 75 μm; 2 μm particle size) at a flow rate of 200 nL/min using a gradient of mobile phase B (0.1% [v/v] formic acid in acetonitrile, LiChrosolv, Merck KGaA) from 1 to 28% B (Curve 4) over 42 min, 28 to 99% B (Curve 6) over 8 min, 99% B for 5 min and back to 1% B over 1 min and equilibrated for 9 min, with a total chromatographic run time of 65 min. Electrospray was achieved with a stainless- steel emitter (40 mm, 1/3200 OD) in the positive ion mode with a spray voltage 1.6 kV. Capillary temperature was set at 250°C. MS data were acquired in a data-dependent manner, with full scan MS spectra (300–1650 *m/z*, (R = 140,000 @ 200 *m/z*) followed by the fragmentation of the top 10 most abundant precursor ions. A lock mass of 445.1200 *m/z*, corresponding to background polysiloxane was used. An inclusion list for the m/z of the two peptides; Ser-Met(oxidized)-Ile-Arg-Pro-Pro-Tyr, 440.2233 *m/z* and Ser-Ile-Arg-Pro-Pro-Tyr-Pro-Ser-Tyr, 540.2796 *m/z*, was used. Dynamic exclusion was set to 45 s, with charge exclusion set for unassigned and singly charged species. Automatic gain control (AGC) target was set to 1 × 10^6^ with a maximum injection time of 20 ms for full scan. Fragmentation of precursor ions was performed by higher-energy collisional dissociation (HCD) with a normalized stepped collision energy of 20, 25, and 30, with a default charge state of 2. MS/MS scans (R = 17,500 @ 200 m/z) were performed with an AGC target value of 1 × 10^5^ and a maximum injection time of 120 ms using an isolation window of 2.2 *m/z*.

The Raw files were searched against the human proteome database (SwissProt UP000005640_9606, 11/08/22) with MaxQuant (v 2.1.4.0) using default parameters except for: Variable modifications: Acetyl (Protein N-term), Deamidation (NQ), Phospho (STY), Oxidation (MP), no Fixed modifications, digestion was set to "Unspecific", Min. peptide length for unspecific search was set to 7. The reconstructed ion chromatogram for two peptides, Ser-Met(oxidized)-Ile-Arg-Pro-Pro-Tyr from AMELY and Ser-Ile-Arg-Pro-Pro-Tyr-Pro-Ser-Tyr from AMELX, were used to assess sex. These two peptides were targeted as they share similar ion intensities and have previously shown to be chromatographically identifiable. Many AMELX peptides have greater ion intensities than AMELY peptides, presumably because the expression of the AMELY protein has the potential to be 10% that of the AMELX [81]. It is therefore important to select peptides that share similar ion intensities when using a qualitative, data-dependent method for analysis.
