# Supplementary material for: Vitamin D status in post-medieval Northern England: Insights from dental histology and enamel peptide analysis at Coach Lane, North Shields (AD 1711–1857)
Source: PLoS One. 2024 Jan 31;19(1):e0296203. doi: 10.1371/journal.pone.0296203 (PMC10830048; doi:10.1371/journal.pone.0296203)
Supplement: S1 Table — (DOCX) [file pone.0296203.s002.docx]

Table 2: Summary of samples collected from teeth from eight non-adults and two adults for enamel peptide analysis. *adult individual.

| **Individual** | **Tooth Sampled** | **Appearance** |
| --- | --- | --- |
| CL 13 | 26 | White, mineralized, roots still forming. |
| CL 21 | 26 | Brown, enamel formed, still mineralizing, no root formation (crown only). |
| CL 25 | 16 | Tan, enamel mineralizing, no roots (crown only). |
| CL 84 | 55 | White, mineralized, roots still forming. |
| CL 118 | 74 | Tan-white, mineralized, roots still forming. Cavity on mesio-buccal side. |
| CL 122 | 27 | White, mineralized, roots formed. |
| CL 127 | 17 | White, mineralized, roots formed. |
| CL 152 | 24 | Tan, enamel mineralizing, root still forming. |
| CL 167* | 35 | White, fully mineralized, no pathology observed, vertical crack on labial side of the enamel |
| CL 253* | 12 | Brown staining (taphonomic), fully mineralized, occlusal wear (exposed dentin) and caries on mesial and distal sides of enamel. |
